# Supplementary material for: Cardiopulmonary Exercise Testing in the Age of New Heart Failure Therapies: Still a Powerful Tool?
Source: Biomedicines. 2023 Aug 6;11(8):2208. doi: 10.3390/biomedicines11082208 (PMC10452308; doi:10.3390/biomedicines11082208)
Supplement: Supplementary file 1 [file biomedicines-11-02208-s001.zip › biomedicines-2510215-supplementary.pdf]

Supplementary Materials

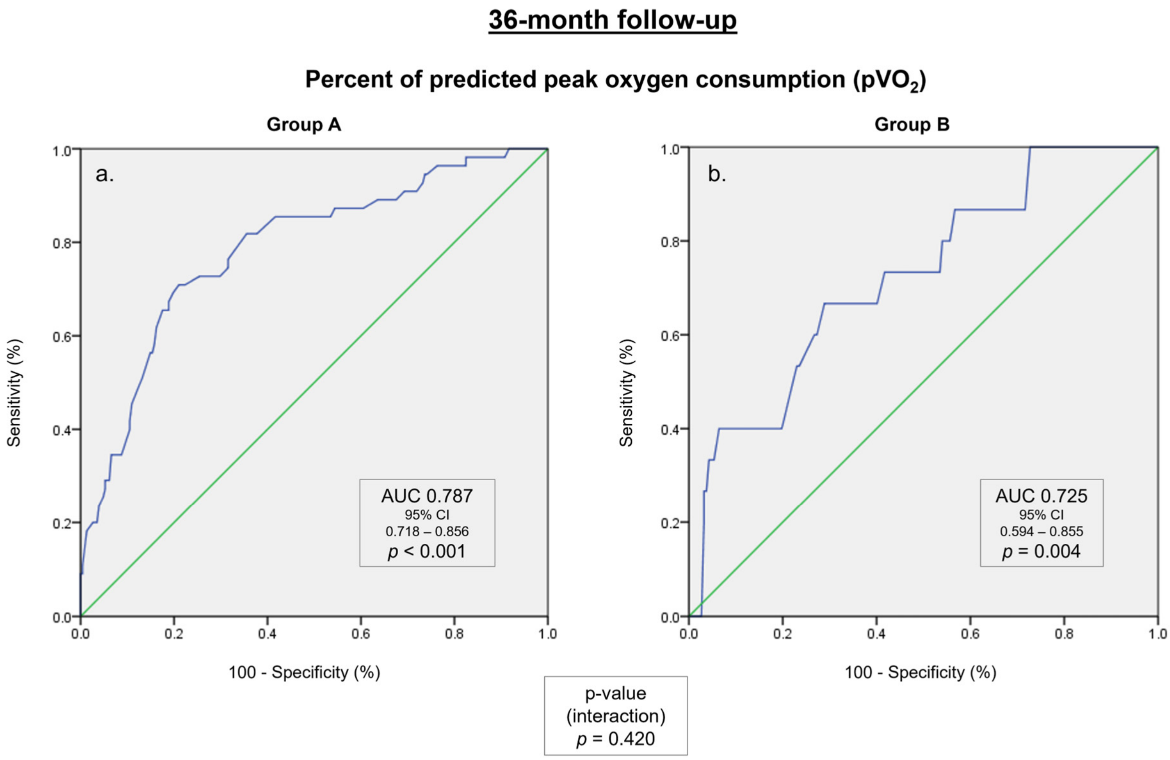

Figure S1. ROC curves for the composite endpoint in a 36-month follow-up. (a) Percent of predicted peak oxygen consumption (pVO_2) in group A. (b) Percent of predicted pVO_2 in group B.

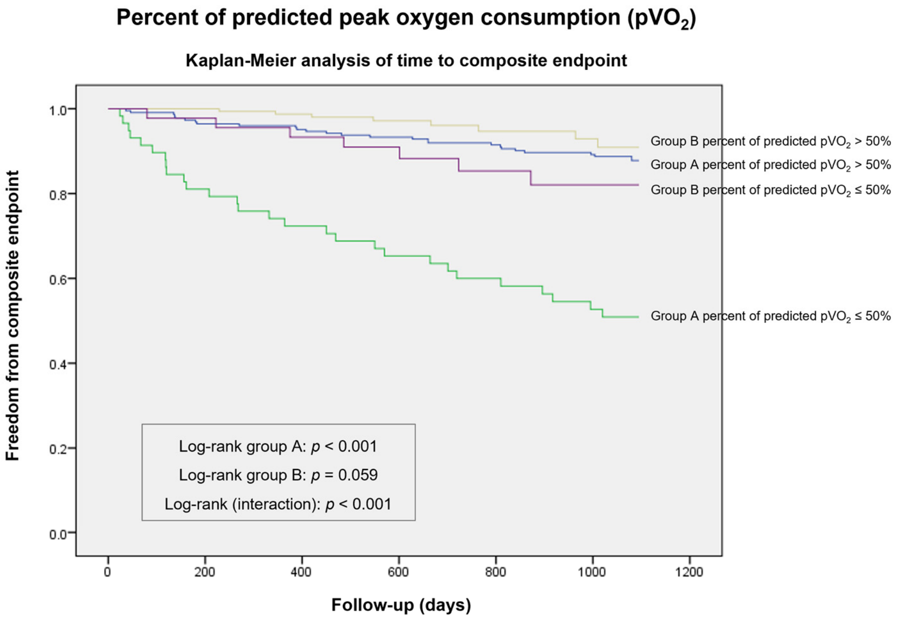

No. at risk							
Group A percent of predicted pVO_2 ≤ 50%	58	47	42	38	35	31	30
Group A percent of predicted pVO_2 > 50%	225	217	214	210	206	201	198
Group B percent of predicted pVO_2 ≤ 50%	45	44	42	41	39	38	38
Group B percent of predicted pVO_2 > 50%	159	159	157	155	153	152	151

Figure S2. Kaplan–Meier survival analysis for the composite endpoint in a 36-month follow-up stratified according to the International Society for Heart and Lung Transplantation (ISHLT)-recommended threshold of percent of predicted peak oxygen consumption (pVO_2) ≤50% in group A and group B.

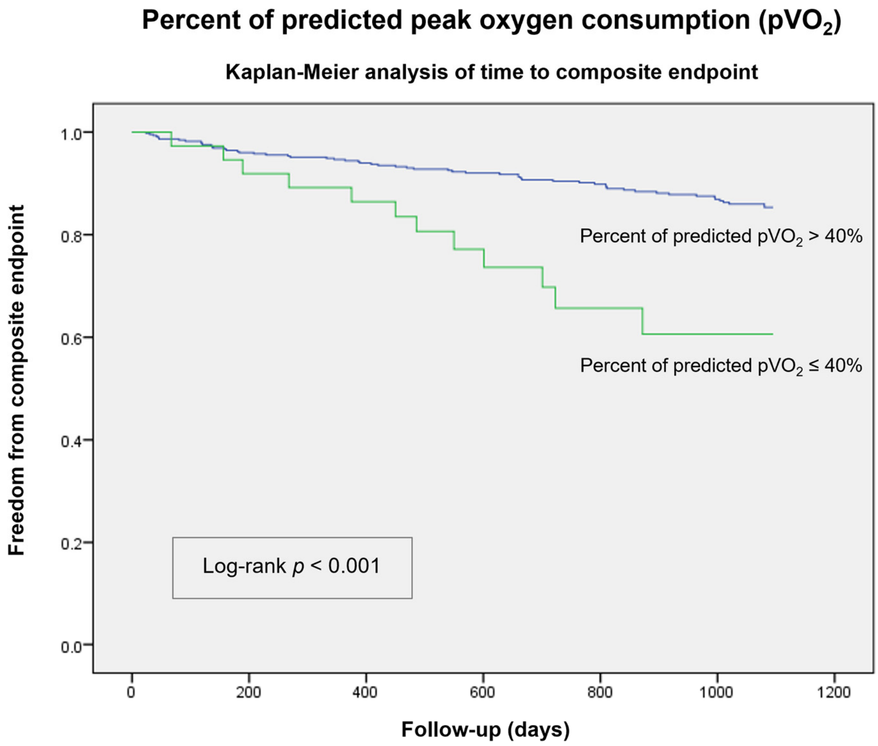

No. at risk

Percent of predicted $pVO_2 \leq 40\%$	37	34	32	29	26	25	25
Percent of predicted $pVO_2 > 40\%$	450	432	423	415	407	397	392

Figure S3. Kaplan–Meier survival analysis for the composite endpoint in a 36-month follow-up in group B stratified according to predicted peak oxygen consumption (pVO2) ≤40%.
